# The Association of Vitamin D Levels and the Frailty Phenotype Among Non-geriatric Dialysis Patients: A Cross-sectional Study

**DOI:** 10.6061/clinics/2018/e116

**Published:** 2018-01-08

**Authors:** Demet Tekdos Demircioglu

**Affiliations:** Physical Medicine and Rehabilitation Department, Istanbul Gelisim University, Istanbul, Turkey

**Keywords:** Frailty, Dialysis, Vitamin D, Deficiency

## Abstract

**OBJECTIVES:**

The aim of this study was to investigate the frequency of frailty and the association of vitamin D levels and the frailty phenotype among non-geriatric dialysis patients.

**METHOD:**

Seventy-four stable, chronic hemodialysis patients from the hemodialysis unit of the hospital were enrolled in the study. The patients' medical histories and laboratory findings were obtained from the medical records of the dialysis unit. Serum parathyroid hormone and 25-hydroxy vitamin D levels were determined using chemiluminometric immunoassays. Frailty was defined by Fried et al. as a phenotype; shrinking, weakness, self-reported exhaustion, decreased activity and slowed walking speed were evaluated.

**RESULTS:**

Forty-one (55%) of the patients were males. The patients were divided into 3 groups according to frailty scores: 39 (53%) patients were frail, 6 (8%) patients were intermediately frail, and 28 (39%) patients were normal. Significant differences were found for 25-hydroxy vitamin D and hemoglobin levels among the groups; however, no differences were observed in body mass index, comorbidities, sex, marital status, education, disease and dialysis durations, or parathyroid hormone, creatinine, serum calcium, phosphorus, and potassium levels.

**CONCLUSIONS:**

Weakness and slowness are serious outcomes of both vitamin D deficiency and frailty, and vitamin D deficiency has been associated with increased risks of decreased physical activity, falls, fractures and death in postmenopausal women and older men. Although studies on frailty have focused on older adults, growing evidence indicates that the frailty phenotype is becoming a factor associated with poor health outcomes in non-geriatric populations with chronic disease.

## INTRODUCTION

The prevalence of frailty syndrome, which is defined as a biological state of increased susceptibility to adverse health conditions, has recently increased from 6.5% to 65%, which is closely related to the aging trends of populations worldwide 1-3.

Chronic kidney disease (CKD) is a major health problem, with a prevalence of 23.4% to 35.8% in persons aged 64 years or older, and affects over 2 million people worldwide 1. CKD increases the risk of cardiovascular disease, frailty and disability 4,5. Although the relationship between CKD and frailty is not completely understood, dysfunction of various systems, including hemoglobin, Interleukin 6 (IL-6), insulin-like growth factor 1 (IGF-1), dehydroepiandrosterone S (DHEA-S), hemoglobuline A1c (Hb A1c), 25-hydroxy vitamin D, vitamin B12, and carotenoids in frailty suggests common pathological pathways for both disorders 1,6.

Frail individuals experience important physical and mental impairments that interfere with activities of daily living 7. The presentation of frailty is variable and may include low physical activity levels and fewer social connections 7,8.

Weakness and slowness are two constituents of frailty that are potential outcomes of vitamin D deficiency. Low vitamin D levels have been associated with increased risks of decreased physical activity, falls, fractures and death in postmenopausal women and older men. Vitamin D deficiency is relatively common in the general population and is also a particular risk for patients with CKD. Reduced sun exposure, impaired production of 25-hydroxy vitamin D, and reduced dietary intake are factors that may affect vitamin D levels, apart from the patient's renal function 9.

Although studies on frailty have focused on older adults, growing evidence indicates that the frailty phenotype is becoming a factor associated with poor health outcomes in non-geriatric populations with chronic disease 10-15.

This study aimed to investigate the frequency of frailty and the association between vitamin D levels and the frailty phenotype among non-geriatric dialysis patients.

## METHODS

This cross-sectional study was conducted in an outpatient hemodialysis clinic between October 1 and November 1, 2016. All stable CKD subjects between 18 and 65 years of age who had received hemodialysis for at least 12 months were invited to participate in the study. Subjects who had received hemodialysis for less than 12 months, had arteriovenous fistulas in both arms, had dementia, were institutionalized for renal or other health problems, or were in preparation for kidney transplantation were excluded from the study.

Hemodialysis therapy was performed three times per week, with each session lasting approximately 4 hours. A standard heparin infusion was administered intravenously before each session, and an erythropoietin dose of 75-150 U/kg/week was given following the session to adjust hemoglobin levels if necessary.

Patients' medical histories and laboratory findings were obtained from the medical records of the dialysis unit. The most recent results of the monthly controlled serum calcium, phosphate, potassium, albumin, hemoglobin, creatinine and alkaline phosphatase levels were recorded. Serum samples were analyzed in an on-site biochemistry laboratory using standard auto-analyzer techniques. Serum intact parathyroid hormone levels were determined using chemiluminometric immunoassays.

Serum 25-hydroxy vitamin D levels were measured using a radioimmunoassay kit (Minividas Biomeriux, France). Vitamin D levels were categorized as risk of deficiency, <12 ng/mL; risk of inadequacy, 12-19 ng/mL; and sufficiency, 20-50 ng/mL 16.

Frailty syndrome was defined by Fried et al. as the presence of at least three of the five phenotypic components, including shrinking, weakness, self-reported exhaustion, decreased activity and slowed walking speed 17. Shrinking was defined as self-reported unintentional weight loss. Patients were questioned about unintentionally losing more than 4.5 kg (of dry weight) during the past year. Weakness was defined as the lowest quintile of the grip strength adjusted for age, sex and BMI, as reported by Massy-Westropp. Handgrip strength was measured 3 times on the non-fistulated side in standing position. A dynamometer (JAMAR Technologies, Inc. Hatfield, PA.) was held at thigh level, and the patient was asked to squeeze the instrument as hard as possible for 3 seconds. The average of the three measurements was used in the analysis. Grip strengths were evaluated based on age- and sex-adjusted values described in the study of Massy-Westropp NM et al. 18.

Self-reported exhaustion was defined as a feeling of generalized weakness or lack of energy in the past 12 months.

The level of activity was evaluated with the Turkish version of The International Physical Activity Questionnaire (IPAQ), which is a self-administered form consisting of seven questions and provides information on sitting, walking, moderate-intensity activities, and the duration of vigorous activity in the last seven days. Physical activity levels were classified as adequate physical activity (3000 MET-min/week), low physical activity (600-3000 MET-min/week) and physically inactive (<600 MET-min/week) 19. *Slowed walking* was evaluated with a 10-meter walk test (10 MWT). A 14-meter-long straight line was drawn, and 2 meters from either end of the line were marked. The test was performed 2 hours before the dialysis session. The time required to walk the 10 meters was recorded, and the average value was calculated after two evaluations 20. Each component of frailty was assigned 1 point when present, and the frailty score was calculated as the sum of the component scores (range 0-5). According to the frailty scores, the subjects were categorized as not frail (score 0-1), intermediately frail (score 2), and frail (score 3-5) 13. The study protocol was approved by the local ethics committee, and written informed consent was obtained from all patients.

### Statistical Analysis

For the statistical analysis, SPSS for Windows Version 16 was used to assess study data. Descriptive statistics are provided as the average and standard deviation; the Pearson correlation coefficient was used to determine correlations, and ANOVA was used to evaluate comparisons. The results were assessed using a 95 % confidence interval with a significance level of p < 0.05.

## RESULTS

A total of 74 hemodialysis patients, 33 (44.6%) of whom were male, aged between 18 and 65 years (mean age±standard deviation, 50.91±10.14 years), were included in the study group.

The mean BMI was 24.89±5.23 kg/m^2^, and the mean disease duration was 9.9±7.2 years. The demographic and clinical characteristics of the patients are summarized in Table 1 and Table 2.

The mean gait speed and IPAQ scores were 1.2±0.67 m/sec and 793.15±832.95 MET/week, respectively. Thirty-eight (51%) of the patients had poor endurance, and 28 (38%) patients reported unintentional weight loss during the past year (Table 3).

A weak positive correlation was observed between the frailty score and age (r=0.244; *p*=0.038).

A negative correlation was found between the frailty score and serum hemoglobin, serum albumin and 25-hydroxy vitamin D levels (r=-0.336, *p*=0.004; r=-0.263, *p*=0.025; r=-0.363, *p*=0.002, respectively). No correlation was observed between the frailty score and BMI, comorbidities, sex, disease and dialysis duration, and parathyroid hormone, creatinine, serum calcium, phosphorus, or potassium levels (Table 4).

The patients were divided into 3 groups according to frailty scores: 39 (53%) patients were frail, 6 (8%) patients were intermediately frail, and 28 (39%) patients were normal. Significant differences were found among groups for 25-hydroxy vitamin D and hemoglobin levels (*p*=0.037, *p*=0.005, respectively); however, no differences were observed in BMI, comorbidities, sex, disease and dialysis duration, and parathyroid hormone, creatinine, serum calcium, phosphorus, and potassium levels (Table 5).

## DISCUSSION

Frail and intermediately frail phenotypes were detected in 53% and 18% of CKD patients, respectively, in the study group. A significant positive correlation was found between the frailty score and age. While frailty was associated with low albumin, hemoglobin and vitamin D levels in this study, the duration of CKD or dialysis treatment and parathyroid hormone levels were not correlated with frailty. The vitamin D and hemoglobin levels were significantly lower in frail subjects.

In a systematic review, the prevalence of frailty ranged between 7% and 42% in pre-dialysis CKD patients 18. Van Munster et al. reported the prevalence of frailty as 44% and 28% in CKD patients older and younger than 65 years of age, respectively 21. The prevalence of frailty increases with age; however, in dialysis patients, frailty may also develop independent of age 13,22-24. In this regard, the detection of frailty in 53 % of the non-geriatric subjects and the correlation between the frailty score and age are noteworthy.

Frailty is more common among patients with comorbidities. Cardiovascular disease, diabetes, stroke, and low albumin concentrations were shown to be significantly associated with frailty in previous studies 25,26. The results of the current study also indicate a correlation between low albumin and hemoglobin levels.

Recent reports suggest that vitamin D deficiency is associated with increased mortality in dialysis patients 27-29. Considering the relationship between frailty and increased mortality, it is relevant to investigate the association between vitamin D levels and frailty. A cohort study on 25-hydoxy vitamin D levels and frailty in elderly women reported that the odds ratio for frailty was higher for those with lower and higher levels of vitamin D at baseline and that non-frail women with low vitamin D levels at baseline were more likely to develop frailty or to die 30. Dreschsler et al. proposed that severe vitamin D deficiency was associated with increased mortality in dialysis patients, and the clinical consequences of vitamin D levels were related to parathyroid hormone levels 31. Vitamin D levels were significantly lower in the frail subjects than in the non-frail subjects in this study group. Although a (negative) correlation between frailty and vitamin D levels was detected, no association was found for parathyroid hormone levels in our study.

Emotional and economic burdens associated with frailty necessitate the prevention or reversal of this process 32. Regular exercise may help maintain or restore functional capacity and independence in the elderly 33 and has been shown to be preventive against frailty and disability in CKD patients. The consequences of decreased physical activity may be reversed, and the survival rate may be improved with physical exercise in both elderly individuals and CKD patients. Frailty is implicated as a common pathway to disability in both aging and CKD, which can be prevented with a multidisciplinary approach, wherein physical exercise is essential 34.

One of the strong aspects of this study is the documentation of the high prevalence of frailty in a limited non-geriatric dialysis patient group. Nevertheless, there are several limitations associated with this study. First, a cross-sectional study design has its own limitations, including response and recall biases and the difficulty of establishing a temporal relationship between the exposures and outcomes. Use of the Fried phenotype may be considered another limitation. Although the frailty phenotype described by Fried et al. has been widely used in previous studies, concerns exist about its efficiency in evaluating the severity of frailty in populations with a high prevalence of frailty 2.

In conclusion, it is important to prevent early-onset disability in end-stage renal disease, which may be accelerated by frailty in this patient population, which includes individuals who already carry a high risk of morbidity and mortality. The constituents of frailty are also potential consequences of vitamin D deficiency, and lower vitamin D levels have been associated with decreased physical performance. It is necessary to identify the underlying factors leading to frailty and their effects on general health and quality of life in CKD patients. Long-term follow-up studies incorporating physical exercise programs will be necessary to establish a place for dialysis patients as active members in society.

## AUTHOR CONTRIBUTIONS

Demircioglu DT designed the study, collected the data, interpreted the results, wrote the manuscript and approved the final version of the manuscript to be published.

## Figures and Tables

**Table 1:** Demographic Characteristics of the Patients.

	Patients (n=74)
**Age (yr; mean ± SD**	50.91±10.14
**Sex**	
** Female (n/%)**	33 (45%)
** Male (n/%)**	41 (55%)
**BMI (kg/m^2^; mean ± SD)**	24.89±5.23
**Married (n/%)**	64 (86%)
**Employed (n/%)**	13 (18%)
**High school graduate (n/%)**	2 (3%)
**Current smoker (n/%)**	14 (19%)

Descriptive statistics were used.

**Table 2:** Clinical Parameters of the Patients.

	Patients (n=74)
**Disease duration (yr; mean ± SD)**	9.9±7.2
**Dialysis duration (yr; mean ± SD)**	8.9±7.1
**Comorbidities: Diabetes (n/%)**	5 (7%)
** Hypertension (n/%)**	17 (27%)
** Coronary arterial disease (n/%)**	23 (31%)
** Cerebrovascular disease (n/%**	3 (4%)
** Peripheral arterial disease (n/%)**	11 (15%)
** Cancer (n/%)**	0 (0 %)
**Serum creatinine**	6.3±2.2
**Serum albumin (mg/dL; mean ± SD)**	4.04±0.41
**Hemoglobin (g/dL; mean ± SD)**	7.04±2.18
**Serum potassium**	5.2±1.02
**Serum calcium**	9.6±0.8
**Serum phosphorus**	5.5±1.54
**Alkaline phosphatase**	76±51
**25 (OH) Vitamin D (ng/dL; mean ± SD)**	13.93±8.64
**Parathyroid hormone (pg/dL; mean ± SD)**	612.16±519.24

**Table 3:** Frailty Phenotypes of the Patients.

	Patients (n=74)
**Weakness (grip strength) (mean ± SD)**	32.66±24.2
**Slowness (gait speed m/s) (mean ± SD)**	1.2±0.67
**Low physical activity (IPAQ scores; MET/week) (mean ± SD)**	793.15±832.95
**Poor endurance (exhaustion) (n/%)**	38 (51%)
**Shrinkage (unintentional weight loss) (n/%)**	28 (38%)
**Frailty scores Frail (n/%)**	39 (53%)
** Intermediately frail (n/%)**	13 (18%)
** Not frail (n/%)**	21 (28%)

**Table 4:** Correlation Coefficients of the Patients.

	Age	Sex	BMI	Disease duration	Dialysis duration	Frailty score	25-hydroxy vitamin D	Hb	PTH	Albumin	Iron	Iron binding capacity
**Age**	1	0.059	0.174	-0.224	-0.217	-0.244^*^	0.147	0.159	-0.063	-0.287^*^	-0.048	0.156
		0.617	0.138	0.058	0.067	0.038	0.215	0.176	0.602	0.013	0.691	0.192
**Sex**	0.059	1	-0.078	-0.176	-0.166	-0.210	-0.095	0.033	0.043	0.107	-0.236^*^	0.058
	0.617		0.511	0.140	0.164	0.075	0.423	0.781	0.720	0.366	0.046	0.626
**BMI**	0.174	-0.078	1	-0.100	-0.108	0.117	0.121	0.195	0.090	-0.171	-0.387^**^	0.282^*^
	0.138	0.511		0.403	0.367	0.323	0.308	0.095	0.454	0.144	0.001	0.017
**Disease duration**	-0.224	-0.176	-0.100	1	0.999^**^	0.084	0.072	-0.359^**^	0.256^*^	0.442^**^	0.101	0.066
	0.058	0.140	0.403		0.000	0.488	0.548	0.002	0.034	0.000	0.406	0.588
**Dialysis duration**	-0.217	-0.166	-0.108	0.999^**^	1	0.078	0.076	-0.349^**^	0.247^*^	0.436^**^	0.094	0.070
	0.067	0.164	0.367	0.000		0.517	0.529	0.003	0.040	0.000	0.437	0.564
**Frailty score**	-0.244^*^	-0.210	0.117	0.084	0.078	1	**-0.363**	-0.336^**^	0.171	0.263^*^	0.036	0.121
	**0.038**	0.075	0.323	0.488	0.517		**0.002**	**0.004**	0.154	**0.025**	0.763	0.313
**25-hydroxy vitamin D**	0.147	-0.095	0.121	0.072	0.076	-0.122	1	0.052	-0.033	0.009	-0.017	-0.068
	0.215	0.423	0.308	0.548	0.529	0.308		0.660	0.785	0.941	0.888	0.572
**Hb**	0.159	0.033	0.195	-0.359^**^	-0.349^**^	-0.336^**^	0.052	1	-0.100	-0.338^**^	-0.346^**^	0.086
	0.176	0.781	0.095	0.002	0.003	0.004	0.660		0.409	0.003	0.003	0.471
**PTH**	-0.063	0.043	0.090	0.256^*^	0.247^*^	0.171	-0.033	-0.100	1	0.344^**^	0.173	-0.137
	0.602	0.720	0.454	0.034	0.040	0.154	0.785	0.409		0.003	0.148	0.255
**Albumin**	-0.287^*^	0.107	-0.171	0.442^**^	0.436^**^	0.263^*^	0.009	-0.338^**^	0.344^**^	1	0.167	0.147
	0.013	0.366	0.144	0.000	0.000	0.025	0.941	0.003	0.003		0.160	0.218
**Iron**	-0.048	-0.236^*^	-0.387^**^	0.101	0.094	0.036	-0.017	-0.346^**^	0.173	0.167	1	-0.370^**^
**Iron binding capacity**	0.156	0.058	0.282^*^	0.066	0.070	0.121	-0.068	0.086	-0.137	0.147	-0.370^**^	1
	0.192	0.626	0.017	0.588	0.564	0.313	0.572	0.471	0.255	0.218	0.001	

PTH: Parathyroid hormone, Hb: Hemoglobin The Pearson correlation test was used for statistical analysis. A value of *p* < 0.05 was considered statistically significant.

**Table 5:** Comparison of Clinical and Laboratory Characteristics of the Patients.

		Mean	Std. deviation	95% confidence interval for mean		Minimum	Maximum
				Lower bound	Upper bound		
**Age**	**Normal**	52.61	10.28	48.62	56.59	29	64
	**Intermediate**	54.50	5.89	48.32	60.68	48	61
	**Frail**	49.03	10.49	45.63	52.43	30	65
**BMI**	**Normal**	24.06	5.40	21.96	26.1595	.00	29.69
	**Intermediate**	27.64	3.78	23.67	31.6111	22.04	30.86
	**Frail**	25.22	5.19	23.53	26.9068	18.55	40.79
**Hb**	**Normal**	8.07	2.58	7.07	9.0796	3.69	12.32
	**Intermediate**	5.92	1.39	4.46	7.3895	3.69	7.80
	**Frail**	6.49	1.68	5.95	7.0474	1.94	9.38
**25-hydroxy vitamin D**	**Normal**	17.09	11.70	12.46	21.722	7.8	68.9
	**Intermediate**	13.45	4.67	8.54	18.351	6.5	21.1
	**Frail**	11.58	5.58	9.77	13.391	3.5	26.6
**PTH**	**Normal**	554.67	499.73	352.82	756.519	7.1	2126.0
	**Intermediate**	358.21	137.32	214.10	502.327	174.9	497.7
	**Frail**	689.56	557.93	508.69	870.422	81.2	2126.0
**Albumin**	**Normal**	3.91	0.44	3.74	4.085	2.9	4.5
	**Intermediate**	4.03	0.35	3.66	4.401	3.4	4.4
	**Frail**	4.14	0.38	4.02	4.274	3.4	5.3
**Fe**	**Normal**	72.27	29.41	60.39	84.15	42	138
	**Intermediate**	64.33	40.77	21.55	107.12	33	141
	**Frail**	72.82	30.99	62.77	82.87	28	152

ANOVA was used to evaluate comparisons.
